# Design and validation of a ribosome display library for synthetic nanobody selection

**DOI:** 10.1007/s44307-026-00112-z

**Published:** 2026-05-14

**Authors:** Weijie Gu, Yaning Li, Jingjing Hong, Zhihao Yue, Yudi Zhang, Shuting Fan, Zhaowen Shen, Tingting Li, Dianfan Li

**Affiliations:** 1 Center for Excellence in Molecular Cell Science, Shanghai Institute of Biochemistry and Cell Biology, University of CAS, Chinese Academy of Sciences, 320 Yueyang Road, Shanghai, 200031 China; 2 School of Agriculture and Biotechnology, Sun Yat-Sen University, Shenzhen, China

**Keywords:** Green fluorescence protein, Nanobody selection, Ribosome display, Single-chain antibody, Synthetic nanobody

## Abstract

Single-domain antibodies (nanobodies) are compact, highly engineerable binding scaffolds widely used in structural biology, biotechnology, and therapeutics. When combined with the Legobody toolkit, they enable high-resolution cryo-electron microscopy (cryo-EM) analysis of small membrane proteins. Ribosome display is a powerful method for generating synthetic nanobodies (sybodies) with exceptionally high library diversity. However, existing sybody libraries are incompatible with the Legobody system, necessitating additional subcloning steps that reduce throughput and limit efficiency. Here, we report the rational design of a new ribosome-display-compatible nanobody library, termed S1.0, engineered for intrinsic compatibility with the Legobody platform through modifications in the C-terminal region. Compared to a benchmark sybody library, S1.0 features increased randomization in the complementarity-determining regions CDR1 and CDR3, while exhibiting reduced variability in CDR2. Selection against calmodulin demonstrated comparable efficiency to the benchmark library. Moreover, selection against thermostable green fluorescent protein (TGP) yielded multiple high-affinity sybodies, with nanomolar dissociation constants validated by size-exclusion chromatography and biolayer interferometry. Importantly, the selected sybodies are directly compatible with the Legobody system without requiring further engineering or subcloning. The S1.0 library represents a valuable resource for nanobody discovery and, in particular, provides an efficient route for generating Legobody-compatible binders suitable for structural studies of challenging small proteins by cryo-EM.

**Supplementary Information:**

The online version contains supplementary material available at 10.1007/s44307-026-00112-z.

## Introduction

Nanobodies (single-domain antibodies) (Muyldermans 2021) are compact and highly engineerable (Li et al. 2021; Yao et al. 2021) binding scaffolds that can be efficiently produced (Li et al. 2024) and tailored for diverse applications, including intracellular targeting (Wang et al. 2025), biosensing (e.g., toxin detection) (Guo et al. 2024), and in vivo imaging (Kang et al. 2021; Wang et al. 2025). Clinically, they have emerged as promising therapeutics—exemplified by Caplacizumab (Scully et al. 2019)—with growing applications in cancer (Fan et al. 2023; Papp et al. 2021; Girard et al. 2025; Fang et al. 2022; Mehra et al. 2024; Zhao et al. 2024; Yi et al. 2025), autoimmune disorders (Dörner et al. 2017; Cong et al. 2024; Leeuw et al. 2025), and infectious diseases (Cunningham et al. 2021).

Conventionally, nanobodies are obtained by immunizing camelids (e.g., llamas) or sharks (Pardon et al. 2014). However, this approach is constrained by antigen toxicity, instability, or the need to capture specific conformational states under non-physiological conditions (e.g., low pH, full-liganded states). In vitro selection platforms (Xia et al. 2025; McMahon et al. 2018; Zimmermann et al. 2018; Doshi et al. 2014; Kondo et al. 2021) address these limitations by using synthetic libraries that encode diverse nanobody sequences with randomized CDR regions. Among these, ribosome display (Zimmermann et al. 2018) is particularly attractive due to its ability to access very large library diversities (10^12^—10^13^) in small volumes.

Nanobodies are especially valuable in structural biology, where their ability to recognize conformational epitopes (Li et al. 2021; Yao et al. 2021; Li et al. 2022; 2022) allows stabilization of distinct functional states (Rasmussen et al. 2011; Schoof et al. 2020; Redrado-Hernández et al. 2024; Irobalieva et al. 2023; Kirchhofer et al. 2010) and facilitates structure determination. In addition, nanobodies can act as crystallization chaperones to facilitate crystal packing (Löw et al. 2013), which is particularly valuable for membrane proteins with limited exposed surface area. They can also serve as fiducial markers to aid image alignment in cryo-electron microscopy (cryo-EM) data processing of membrane proteins that lack discernible soluble regions (Yang et al. 2022).

To further improve the visualization of small membrane proteins, the Legobody system (~ 120 kDa) has been developed to increase the effective molecular weight of nanobody–target complexes (Wu and Rapoport 2021; Kang and Chen 2023; Lin et al. 2025). However, the utility of this approach is currently limited by incompatibility with the widely used Seeger ribosome display libraries (Zimmermann et al. 2018; Ackle et al. 2025), whose nanobody constructs lack the C-terminal His-tag required for Legobody binding. As a result, additional subcloning steps are needed, creating a bottleneck for high-throughput screening.

Here, we sought to address this limitation by designing a ribosome display nanobody library that is intrinsically compatible with Legobody assembly. Specifically, we incorporated a C-terminal His-tag into the library scaffold with redesigned sequence diversity with an aim to shift binding paratope towards the CDR3/1 region. We performed sybody selection against a thermostable green fluorescent protein (TGP) and calmodulin, demonstrating that the modified library supports efficient binder discovery. We also demonstrate that the sybodies bind to Legobody without re-engineering. This library should provide a useful source not only for nanobody discovery, but also for selection of Legobody-compatible nanobodies for structural studies.

## Materials and methods

### Library construction

DNA encoding a nanobody having the most frequent residues in the CDR region (see text for details) was synthesized and subcloned into a pRDV5 vector (Li et al. 2024). This plasmid, dubbed pRDV5-Nb, was used as a template to amplify the following three fragments using Phusion High-Fidelity DNA polymerase (Cat. M0530L, New England Biolabs) or PrimeSTAR Max DNA Polymerase (Cat. R045A, Takara). Fragment 1 containing the T7 promoter, ribosome binding site, and Framework 1 of the nanobody (nanobodies contain 4 framework regions spaced by three CDR regions) was amplified using the primer pair 5’-Flank-F (5’-CGAAATTAATACGACTCACTATAGGGAGAC-3’) and FW1-R (5’-CCCTGACGCTGCGCATGATAA C-3’). Fragment 2 containing Framework 3 was amplified using the primer pair FW3-F (5’-TACGCTGATAGCGTGAAAGG-3’) and FW3-R (5’-ATGCATGGTCTCACAGCTGTATCTTCTGGTTTTAATGAG-3’). Fragment 3 containing Framework 4, a short linker for legobody (Wu and Rapoport 2021) assembly (SLEHHHHHH), the tolA sequence (for ribosome stalling), and a *BspQ*I cleavage site, was amplified using the primer pair FW4-F (5’-GGCCAGGGTACTCAAGTCAC-3’) and TolA-R1 (5’-CAGCAGCTATAGCTCTTCAAAACCGCACACCAGTAAGGTGTGCGGTTTCAGTTGCCGCTTTCTTTCTTG-3’). The fragments were gel-purified (Cat. 28706, Qiagen) and used as megaprimers in the following assembly steps.

Single-strand DNA (ssDNA) containing desired trinucleotides according to our design in the CDR and juxta-CDR regions were custom-ordered from GeneScript. These ssDNA, dubbed as rCDR1, rCDR2, rCDR3, respectively, contained semi-randomized CDR1/2/3 sequences and flanking regions that overlap with the three fragments mentioned above. A PCR reaction was employed to assemble Fragment 1 with ssDNA rCDR1 by three primers to yield a fragment named F-CDR1. The 5’-primer anneals to the 5’-end of Fragment 1 (5’-Flank-F). The first 3’-primer, named FW2-R (5’-ATGCATGGTCTCACCCATTCGCGCTCCTTCCCTGGAGCTTGACGAAACC-3’), anneals to the 3’-end of rCDR1 with a long extension to introduce Framework 2 and a Type IIS restriction site for ligation later. A second 3’-primer, named Link1-R, is the truncated version of FW2-R that share the 21 nucleotide of the 3’-end of FW2-R. During the PCR reaction, Primer 5’-Flank-F and Link1-R were included at a final concentration of 1 μM, while the FW2-R primer was included at 25 nM. Fragment 1 and rCDR1 were included at a final concentration of 50 nM and 25 nM respectively.

Fragment 2 and rCDR2 were joined together to yield a fragment named F-CDR2 using primer pairs (1 μM each) Link1-F (5’-GAATGGGTGAGACCATGCATGAAGACCTTGGGTTGC-3’) and FW3-R. Fragment 2 and rCDR2 were included at a final concentration of 50 and 25 nM respectively.

Fragment 3 and rCDR3 were joined together to yield a fragment named F-CDR3 using primer pairs (1 μM each) Link2-F (5’-GTGAGACCATGCATTATATCGAAGACCTGCTGTTTACTACTG-3’) and TolA-R2 (5’-CAGCAGCTATAGCTCTTCAAAACC-3’). Fragment 3 and rCDR3 were included at a final concentration of 50 and 25 nM respectively.

F-CDR1, F-CDR2, and F-CDR3 were analyzed by 3% (w/v) agarose gel to assess purity. DNA fragments were either purified using a PCR-clean kit (F-CDR3) or gel-purified (the other two). F-CDR1 (2 μg) and F-CDR2 (2 μg) were separately digested with *Bsa*I and *Bbs*I respectively, gel-purified, and ligated to yield 1.2 μg of a fragment named CDR1—2. A PCR reaction using 300 ng of this fragment and 0.8 μM each of the primer pair 5’-Flank-F and FW3-R was performed to amplify the fragment, yielding 75 μg of CDR1—2. CDR1—2 (65 μg) was digested using *Bsa*I again, purified and ligated with F-CDR3 (68 μg) which was digested using *Bbs*I, yielding 11 μg of DNA products that contain all the functional units (T7 promoter, Ribosome binding site, nanobody-encoding open reading frame, a spacer, and TolA for ribosome stalling). Finally, a 5-mL PCR reaction (PrimeSTAR Max DNA Polymerase (Cat. R045A, Takara) was performed using 10 μg of the fragment as the template and the primer pair 5’-Flank-F and TolA-R2 yielding 92 μg of DNA. Fifty micrograms of the DNA were digested with *BspQ*I to remove an unintentional DNA fragment that contained an approximate 200—bp sequence reverse complement to the spacer region. Gel purification was performed to yield 48 μg of DNA.

DNA (10 μg) was transcripted using the RiboMAX™ Large Scale RNA Production Systems (Cat. P1300, Promega). The product was purified using RNeasy Mid Kit (Cat. 75144, QIAGEN) to yield 360 μg mRNA in a total volume of 200 μL. The mRNA products were stored at −80 °C as 2.7-μL aliquots.

### Expression and purification of TGP

The thermostable green fluorescence protein (TGP) (Cai et al. 2020) was expressed in *Escherichia coli* as a C terminally His- tagged protein. BL21 (DE3) cells carrying pETSG (Cai et al. 2019) were induced at OD_600_ of 0.6–0.8 for 18 h at 20 °C using 1 mM isopropyl-β-_D_-thiogalactopyranoside (IPTG). Cells were harvested by centrifugation and resuspended in a lysis buffer containing 150 mM NaCl, 50 mM Tris–HCl pH 8.0, followed by cell disruption using a high-pressure homogenizer. The lysate was clarified by centrifuging at 20,000 × g for 30 min at 4 °C. The supernatant was incubated with 1 mL of Ni–NTA resin for 2 h with gentle agitation at 4 °C. The resins were loaded into a gravity column and washed with 20 column volume (CV) Wash buffer (20 mM imidazole in lysis buffer). TGP was eluted with 300 mM imidazole.

### Expression and purification of calmodulin

The coding sequence of calmodulin (Uniprot ID P62162) was synthesized (Azenta), digested with *BamH*I/*Kpn*I and ligated into vector p3EC (Cai et al. 2019) as a C-terminally His tagged protein. BL21(DE3) cells carrying the plasmid were induced at an OD₆₀₀ of 0.6–0.8 with 1 mM IPTG and grown for 18 h at 20 °C. Cells were harvested by centrifugation, resuspended in lysis buffer (150 mM NaCl, 50 mM Tris–HCl pH 8.0, 10 mM CaCl₂), and disrupted by sonication on ice. The lysate was clarified by centrifugation at 48,000 × g for 30 min at 4 °C. The supernatant was incubated with 1 mL of Ni–NTA resin for 2 h at 4 °C with gentle agitation. The resins were loaded into a gravity column and washed with 20 CV of wash buffer containing 20 mM imidazole in lysis buffer. Calmodulin was eluted with 300 mM imidazole.

### Expression and purification of sybodies

Sybodies, which contains a hexa-histidine tag from the library design, were expressed in *E. coli* with an additional Myc and His-tag at the C-terminus that were encoded by the vector pSb-init (Zimmermann et al. 2018). Overnight culture of MC1061 cells carrying the plasmids was seeded into 50 mL terrific broth (TB) supplemented with 25 μg/mL chloramphenicol at a 1:100 ratio. Cells were cultured in a 37 °C-incubator for 2 h. The temperature was then dropped to 22 °C to slow down cell growth. Cells were induced with 0.02%(w/v) arabinose at OD_600_ of 0.6—0.8 for 17 h. Cells were harvested by centrifugation and resuspended with 1 mL TES buffer (20%(w/v) sucrose, 0.5 mM EDTA, and 200 mM Tris–HCl pH 8.0) and rotated at 4 °C for 30 min for dehydration. Dehydrated cells were disrupted by rehydration with 2 mL ice-cold MilliQ H_2_O for 1 h at 4 °C. Periplasm extractions were harvested by centrifuging at 20,000 × g for 30 min at 4 °C. The supernatant was adjusted to contain 150 mM NaCl, 5 mM MgCl_2_ and 20 mM imidazole before being mixed with 0.2 mL Ni–NTA resins at 4 °C for 1 h with gently rotation. The resins were packed into a gravity column and washed with 20 CV Wash buffer (20 mM imidazole, 150 mM NaCl, 20 mM Tris–HCl pH 8.0), before being eluted with 300 mM imidazole.

### Expression and purification of Fab_8D3_2

The genes encoding the light chain and the C terminally His tagged heavy chain of Fab_8D3_2 (Wu and Rapoport 2021) were inserted into the pDEC vector respectively (Li et al. 2021; 2024). Expi293 cells at 2.0 × 10^6^ per milliliter were transfected with both plasmids, each at a final concentration of 0.5 mg/L, using linear polyethylenimine (3 mg/L). To enhance expression, sodium butyrate was added to a final concentration of 10 mM between 10 and 12 h post transfection. The cells were then cultured in a flask for an additional 60 h. The secreted Fab was harvested from the culture medium by centrifugation followed by filtration through a 0.22 μm filter. The resulting supernatant was adjusted to contain 5 mM imidazole, 150 mM NaCl, and 50 mM Tris–HCl pH 8.0, then incubated overnight with Ni Sepharose (Cat. 17371202, Cytiva). The mixture was packed into a gravity column, washed with 20 CV of Buffer A (150 mM NaCl, 20 mM Tris–HCl pH 8.0) supplemented with 10 mM imidazole, and finally eluted with 300 mM imidazole in Buffer A. The eluate was aliquoted, snap frozen in liquid nitrogen, and stored at –80 °C until use.

### Expression and purification of MBP_PrAC

The DNA fragment encoding the maltose-binding protein (MBP) in fusion with Protein A/C (MBP_PrAC) (Wu and Rapoport 2021) with a C-terminal 8 × His tag was inserted into the p3EC (Cai et al. 2019) vector. *E. coli* BL21(DE3) cells harboring this plasmid were cultured in LB broth containing 50 μg/mL kanamycin at 37 °C in a shaking incubator (220 rpm) until the OD_600_ reached 0.6. Cells were induced with 1 mM IPTG for 4 h at 37 °C. The bacterial pellet was collected, re-suspended in Lysis Buffer (400 mM NaCl, 20 mM imidazole, 50 mM HEPES pH 7.5) supplemented with 5 mM MgCl₂, 10 μg/mL DNase I, and 1 mM PMSF, and then disrupted with a homogenizer. After centrifugation at 25,000 × g for 30 min to remove cell debris, the cleared lysate was mixed with Ni–NTA resins pre-equilibrated with Lysis Buffer. Following batch binding at 4 °C for 3 h, the resin beads were washed with 20 CV of Lysis Buffer and subsequently eluted with 300 mM imidazole in Buffer B (150 mM NaCl, 20 mM HEPES pH 7.5). The eluate was further purified by size exclusion chromatography on a Superdex 200 Increase 10/300 GL column with Buffer B containing 5% (v/v) glycerol. Fractions of the peak were collected, aliquoted, snap frozen in liquid nitrogen, and stored at –80 °C before use.

### Pull-down of Legobody and TGP

TGP was mixed with Legobody components at a molar ratio of TGP: sybody: MBP_PrAC and Fab_8D3_2 = 2:1.5:1:1.5 and incubated on ice for 1 h. The mixture was incubated with amylose resin for 1 h at 4 °C with gentle rotation. The resins were washed with 10 CV of Buffer C (150 mM NaCl, 50 mM Tris–HCl pH 8.0) to remove unbound proteins and then eluted with 20 mM maltose in Buffer C. The elution was analyzed using SDS-PAGE to check complex formation.

### Biotinylation of TGP and calmodulin

To facilitate sybody selection, an Avi tag (GLNDIFEAQKIEWHE) was fused to the C-terminus of TGP or calmodulin. The resulting construct (TGP_avi_/calmodulin_avi_) was expressed and purified using the same procedure described above.

For biotinylation, 1 mL purified TGP_avi_/calmodulin_avi_ at 3 mg/mL was incubated with 0.17 mg/mLof biotin ligase BirA, 5 mM ATP, 10 mM magnesium acetate, 0.16 mM biotin and incubated at 4 °C for 16 h. The biotinylated TGP_avi_ and calmodulin_avi_ was fractionized by gel filtration on a Superdex 200 10/300 GL column with a mobile phase containing 150 mM NaCl, 20 mM Tris–HCl pH 8.0. For TGP_avi_, the fractions with absorbance at 493 nm (A_493_) were pooled together and the concentration was determined using A493 with the molar extinction coefficient of 64,500 M^−1^ cm^−1^. The concentration of calmodulin_avi_ was determined using A280 with the molar extinction coefficient of 2,980 M⁻^1^ cm⁻^1^. The pooled fractions were aliquoted, flash-frozen with liquid nitrogen, and stored at −80 °C before use.

### Sybody selection

The sybody selection process consisted of ribosome display, phage display, and single-colony screening. An in vitro translation reaction containing 4.8 μg mRNA was carried out using the PUREfrex 2.1 kit (Cat. PF213—0.25-EX, Genefrontier) supplemented with disulfide bond isomerase DsbC (DS supplement, Cat. PF005—0.5-EX, Genefrontier), according to the manufacturer’s instructions. The reaction mix was diluted into 100 μL ice-cold panning solution containing 150 mM NaCl, 50 mM magnesium acetate, 0.5% (w/v) BSA, 0.1% (w/v) Tween 20, 0.5% (w/v) heparin, 1 μL RNaseIn (RNase inhibitor; Cat. N2611, Promega), and 50 mM Tris acetate pH 7.4, followed by centrifugation at 20,000 × g for 5 min at 4 °C. The supernatant was incubated with 50 nM biotinylated TGP_avi_/calmodulin_avi_ on ice for 20 min. A pull-down step was performed by adding 12 uL streptavidin beads (Dynabeads MyOne Streptavidin T1; Cat. 65601, Invitrogen) to the solution. mRNA was eluted from the beads with EDTA and yeast RNA. Recovered mRNA was reverse transcribed using a primer (5′-CTTCAGTTGCCGCTTTCTTTCTTG-3′). The resulting cDNA library was amplified by PCR using a primer pair with the following sequence: 5′-ATATGCTCTTCTAGTCAAGTACAGTTAGTAGAAAGTGGTGGTGG-3′ and 5′-TATAGCTCTTCATGCTGCGCTATGATGATGATGATGATGCTC-3′. The PCR products were gel-purified, digested with *BspQ*I and ligated into the pDX-init vector digested with the same enzyme.

The ligation products were transformed into *E. coli* SS320 competent cells by electroporation. Phage particles were generated and panned as described earlier (Li et al. 2021). Briefly, the first round of phage display was performed by incubating 4.7 mL of phage particles premixed with 50 nM biotinylated TGP_avi_/calmodulin_avi_ in a total of 47 wells of a 96-well plate coated with 60 nM neutravidin (Cat. 31000, Thermo Fisher Scientific). The bound phage particles eluted using trypsin were amplified, and used for the second round of phage display. To this end, 100 μL of phage particles preincubated with 50 nM of biotinylated TGP_avi_/calmodulin_avi_ were mixed with 12 μL of Streptavidin C1 beads (Dynabeads MyOne Streptavidin C1; Cat. 65001, Invitrogen). After challenging with 5 μM of non-biotinylated TGP_avi_/calmodulin_avi_ to remove high off-rate binders, the phage particles were recovered to infect *E. coli* SS320 cells. All the panning procedure was conducted in the buffer containing 0.1%(w/v) Tween 20, 150 mM NaCl, 50 mM Tris–HCl pH 7.4. The enriched sybody library contained in phagemid was subcloned into pSb-init vector using fragment-exchange (FX) cloning and transformed into *E. coli* MC1061 for single-colony expression and purification.

### Enzyme-linked immunosorbent assay (ELISA)

To screen TGP binders at a single-colony level, sybodies were expressed and extracted as follows. MC1061 single colonies carrying pSb-init-sybody plasmids were inoculated into 1 mL TB medium supplemented with 25 μg/mL chloramphenicol in a 2.2 mL 96-well plate. Cells were cultured at 37 °C, 300 rpm for 5 h and then inoculated into a new 96-well plate by a dilution factor of 1:20. After growing at 37 °C for 2 h, the temperature was dropped to 22 °C and the cells were cultured for another 1.5 h before induced with 0.02%(w/v) arabinose. After 16 h, cells were harvested by centrifugation at 3,345 × g for 30 min. Cell pellets were resuspended with 100 μL buffer containing 0.5 μg/mL lysozyme, 20%(w/v) sucrose, 0.5 mM EDTA, and 50 mM Tris–HCl pH 8.0 with shaking at room temperature for 30 min. The suspension was diluted with 900 μL buffer containing 150 mM NaCl, 1 mM MgCl_2_, and 50 mM Tris–HCl pH 7.4. Periplasm extracts were harvested by centrifugation at 3,345 × g for 30 min at 4 °C. The mixture was centrifuged again at 3,000 × g for 30 min at 4 °C, and the resulting supernatant fraction was used directly for ELISA or fluorescence detection size exclusion chromatography (FSEC) assay.

For ELISA, 5 μg/mL protein A diluted in PBS buffer was incubated with F96 MaxiSorp nunc-immuno plate (Cat. 442404, Thermo Scientific) plates at 4 °C overnight. The plates were washed once with 125 μL TBS (150 mM NaCl, 20 mM Tris–HCl pH 7.4) per well and blocked with 0.5%(w/v) BSA in TBS buffer at room temperature (RT) for 30 min. The plates were washed three times with TBS before binding with anti-Myc antibodies (Cat. M4439, Sigma) diluted in TBS-BSA-T buffer (TBS supplemented with 0.5% (w/v) BSA and 0.05% (v/v) Tween 20) as 1:2,000. After 20 min, the plates were washed three times with TBS-T (0.05% (v/v) Tween 20 in TBS). The above-mentioned Myc-tagged sybody extraction was added and incubated at RT for 20 min. After washing three times with TBS-T, biotinylated calmodulin or TGP (as control) was added to each well to a final concentration of 50 nM. After 20 min incubation, the plates were washed three times with TBS-T and incubated with streptavidin-HRP conjugate (Cat. S2438, Sigma) diluted in TBS-BSA-T buffer as 1:5,000. After incubation at RT for 20 min, the plates were washed three times with TBS-T and signal was developed with 50 μL developing buffer (51 mM Na_2_HPO_4_, 24 mM citric acid, 0.006% (v/v) H_2_O_2_, 0.1 mg/mL 3,3’,5,5’-tetramethylbenzidine). ELISA signal (absorbance at 650 nm) was measured with a plate microplate reader.

### FSEC assay to screen TGP binders

For FSEC assay, 50 μL above-mentioned sybody periplasmic extracts were mixed with 0.25 μM of TGP and incubated on ice for 30 min. One microliter of the mixture was loaded onto a Sepax Zenix-C SEC-300 column connected to a Shimadzu high-performance liquid chromatography (HPLC) machine equipped with a fluorescence detector (RF-20A, Shimadzu) with excitation/emission wavelength of 482/508 nm. The running buffer contained 150 mM NaCl and 50 mM Tris–HCl pH 8.0. A TGP sample with an irrelevant sybody was used as a negative control.

### Biolayer interferometry assay

Binding kinetics between TGP and sybody were determined using biolayer interferometry (BLI) with an Octet system. Biotinylated TGP was immobilized onto a streptavidin-coated sensor ( Cat 18—5019, ForteBio) by incubating with 1 μg/mL TGP_avi_ diluted in the Blank Buffer containing 0.005% Tween20, 150 mM NaCl, 50 mM Tris pH 8.0. The sensors were incubated with the Blank Buffer for baseline equilibration. The sensors were then soaked in a well containing sybody at various concentrations in the Blank Buffer for association, followed by incubation in the Blank Buffer for dissociation. All kinetic data were processed and globally fitted using Octet Analysis Studio 13.0.3.52 (Sartorius) with the 1:1 binding model which assumes a reversible bimolecular interaction between the immobilized antigen and analyte (Sybody). The fitting is based on the following equation: d[AB]/d*t* = *k*_on_ × [A](*t*) × [B] − *k*_off_ × [AB](*t*), [AB](0) = 0, where [AB](*t*) is molar concentration of complex at the interaction surface at time *t*; [A](*t*) is the molar concentration of analyte at the interaction surface at time *t*; [B] is molar concentration of available immobilized ligand; *k*_on_ is association rate constant (M^−1^ s^−1^); *k*_off_ is dissociation rate constant (s^−1^).

### Next-generation-sequencing (NGS) sample preparation and sequencing

To generate the adaptor sequences and barcode for NGS sequencing, 20 ng naïve DNA library was used as PCR template. The first PCR was performed with the primer pair P5-F (5’-GACACTCTTTCCCTACACGACGCTCTTCCGATCTATATCCATGGGTAGTCAAGTACAG-3’) and P7-R (5’-CATGGTGACTGGAGTTCAGACGTGTGCTCTTCCGATCTGGCCATATAAAGCTTTGCG-3’). The second PCR was performed using the primer pair P5-F2 (5’-AATGATACGGCGACCACCGAGATCTACACCTCCATCGAGACACTCTTTCCCTACACG-3’) and P7-R2 (5’-CAAGCAGAAGACGGCATACGAGATTTCTCGCATGGTGACTGGAGTTCAG-3’). The resulting fragments were gel-purified (Cat. 740609, Macherey Nagel) and sequenced using an Illumina NovaSeq X Plus system in paired-end 150 bp (PE150) mode.

### NGS data processing

Raw Illumina NovaSeq X Plus PE150 reads were quality-filtered and adapter-trimmed with fastp (Chen et al. 2018). The sybody sequences were annotated with custom library built by the original nanobody template using MIXCR v3.0.3. Clonotype assembly was carried out based on CDR1 and CDR3 regions. The sequence information was extracted for downstream plotting and analysis.

## Results

### Design and validation of a synthetic nanobody library

In principle, a binder is guaranteed given unlimited chemical space, such as the all possible combinations across the three CDRs. However, the practical diversity of even the largest display platforms (e.g., mRNA display at 10^13^–10^14^) is vastly exceeded by the theoretical diversity of a fully randomized library. For instance, a library with just 15 fully randomized residues has a theoretical diversity of ~ 3.3 × 10^19^. Therefore, semi-randomization strategies are essential to create manageable library sizes while maximizing meaningful diversity. This involves selectively randomizing each CDR position with tailored amino acid preferences.

Our design was informed by our previous successful experience screening synthetic nanobodies (sybodies) from the Seeger Concave library (Zimmermann et al. 2018; Yue et al. 2024; Li et al. n.d.). This library features a short, 7-residue CDR3 and is designed with a stable hydrophobic core to form a concave paratope (Zimmermann et al. 2018), ideal for recognizing convex epitopes.

**Fig. 1:**
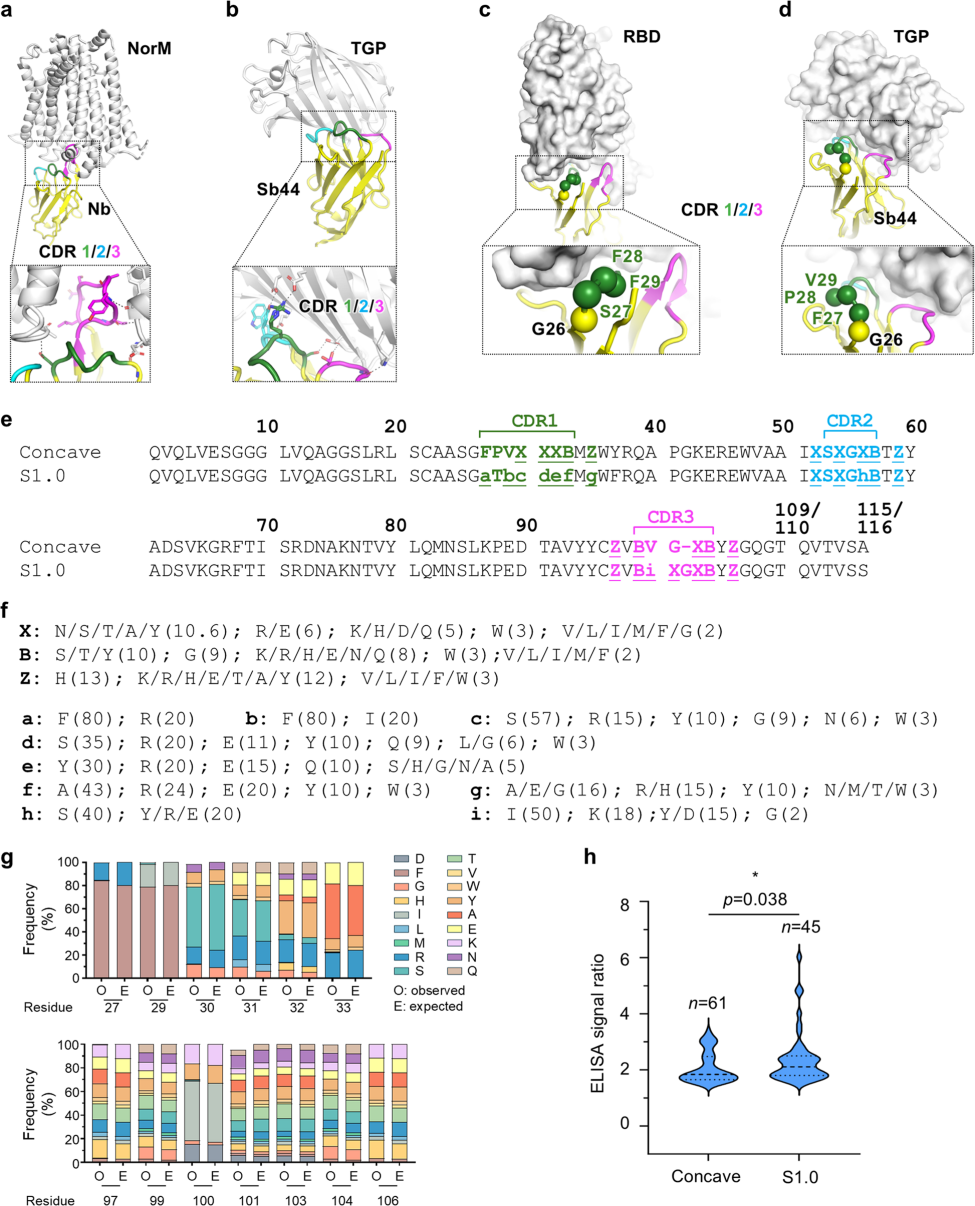
Design and validation of S1.0 library. **a**,**b** Overall structure of antigens (light gray cartoon) and nanobodies (yellow cartoon with colored CDR regions). PDB entries 7PHP (**a**) (Bloch et al. 2021) and 6LZ2 (**b**) (Cai et al. 2020) are used for analyses. Residues involved in antigen–antibody binding are shown as stick representations. Black dashed lines indicate H-bonds or salt bridges within 3.4 Å. **c**,**d** Overall structure of antigen (light gray surface) and nanobodies (yellow cartoon with colored CDR regions). Sphere represents key residues involved in antigen binding. PDB entries 7F5G (**c**) (Li et al. 2022) and 6LZ2 (**d**) (Cai et al. 2020) are used for analyses.** e** Sequence alignment of Concave library and S1.0 library. CDR regions are marked with brackets and with different colors. CDR residues and randomized residues in flanking region were highlighted with bold characters and indicated color. Randomized positions are underlined. Non-capital letters indicate randomized compositions. **f** Amino acid composition of randomized positions in (**e**). **g** Comparison of observed amino acid frequencies from next-generation sequencing (NGS) (O, observed) with the expected design (E). Residues are numbered in accordance with (**e**). **h** Violin plot showing the ELISA signal ratios of potential calmodulin binders. The ratio was calculated as the ELISA signal in the presence of Ca^2^⁺ divided by the signal in the absence of Ca^2^⁺ (with EGTA/EDTA). Only clones with a signal ratio greater than 1.5 are plotted. The upper dotted line, dashed line, and lower dotted line represent the 25th percentile, median, and 75th percentile of the population, respectively. Statistical significance was assessed using an unpaired two-tailed *t*-test

Analysis of concave sybody-antigen complex structures revealed several key features. First, compared to naturally occurring nanobodies, which often depend heavily on CDR3 for antigen recognition (Fig. 1a), the paratope of concave sybodies is shifted toward the CDR1 and CDR2 regions (Fig. 1b). This is likely a direct result of the short CDR3 and its limited randomization in the Seeger library (Zimmermann et al. 2018), where only three core CDR3 residues and two flanking residues are varied. Second, unlike some natural nanobodies (Fig. 1c), the first half of CDR1 in the Concave sybodies do not engage with antigens (Fig. 1d). This is consistent with the original library design, where these flanking and CDR1 residues are fixed (Zimmermann et al. 2018) to maintain the concave architecture.

Based on these observations, we designed a new library (termed S1.0) with modifications to the Seeger (Zimmermann et al. 2018) framework (Fig. 1e) with an aim to shift the paratope towards the CDR3/1 side. First, the CDR3 length was increased by one residue, and the number of randomized positions in the CDR3 and its flanking region was set to seven. Second, the number of randomized residues in the CDR1 and its flanking region was also increased to seven. Third, we reduced the diversity of the CDR2 region by changing one fully (all amino acids except cysteine and proline) randomized position to a limited randomization (Fig. 1e, 1f). This position, observed to face the antigen, was restricted to a small, flexible residue (serine) and three bulky residues (tyrosine, arginine, and glutamate) to diversify surface shape (Fig. 1f).

This S1.0 design included 19 randomized positions. If fully randomized, its theoretical diversity would be ~ 5.2 × 10^24^, far exceeding the practical limit of ribosome display (10^12^–10^13^). Therefore, we implemented limited variability at several positions (Fig. 1f). Generally, bulky residues were enriched at positions expected to be distal from the epitope to increase the likelihood of reaching the antigen, and residue choices were based on natural amino acid frequencies observed in native nanobodies. The final design has a theoretical diversity of 10^18^, comparable to the Seeger Concave library (Zimmermann et al. 2018).

The library was constructed through a series of overlapping PCRs, Type IIS restriction enzyme digestion, and T4 DNA ligation (see Methods). To assess whether the constructed library faithfully captured the designed sequence diversity, we performed next-generation sequencing (NGS) on the naïve S1.0 DNA library, focusing on the CDR1 and CDR3 regions. We obtained approximately 9 × 10^7^ reads, of which 98% were unique sequences.

For the CDR1 region, all 32 designed randomized compositions (Fig. 1e, f) were represented. Of these, 23 compositions showed excellent agreement with the expected distribution (discrepancy < 10%). Two compositions exhibited moderate discrepancies (10%–13%), while the remaining six showed larger discrepancies (24%–55%) (Fig. 1g**, **Table S1). In the CDR3 region, among the 97 designed randomized compositions, 46% (45 compositions) displayed frequencies within 10% discrepancy from the designed values, 34 compositions showed discrepancies between 10%–20%, and 18 compositions showed discrepancies greater than 20% (Fig. 1g**, **Table S1).

Taken together, the S1.0 library largely preserved the expected sequence compositions, demonstrating high overall fidelity to the original design.

### Screening of sybodies against calmodulin

To evaluate the performance of the S1.0 library, we performed sybody selections against calmodulin in parallel using both the S1.0 library and the benchmark Seeger Concave library (Zimmermann et al. 2018). After one round of ribosome display followed by two rounds of phage display, individual clones were screened by ELISA. To select binders specific to the Ca^2^⁺-bound conformation of calmodulin, a parallel ELISA was conducted in the presence of 10 mM EDTA-EGTA. Clones were considered positive if the ELISA signal ratio (Ca^2^⁺ condition/EDTA-EGTA condition) exceeded 1.5.

Ninety-six clones were randomly picked from each library for ELISA evaluation. The S1.0 library yielded 45 positive clones, while the Seeger Concave library yielded 61 positive clones (Fig. 1h**, **Fig. S1). Although the S1.0 library showed a modestly lower positive clone rate, the signal ratios were statistically significantly higher than those from the benchmark library (Fig. 1h). These results demonstrate that the S1.0 library is of comparable quality to the established benchmark library.

### Screening synthetic nanobodies against TGP

**Fig. 2:**
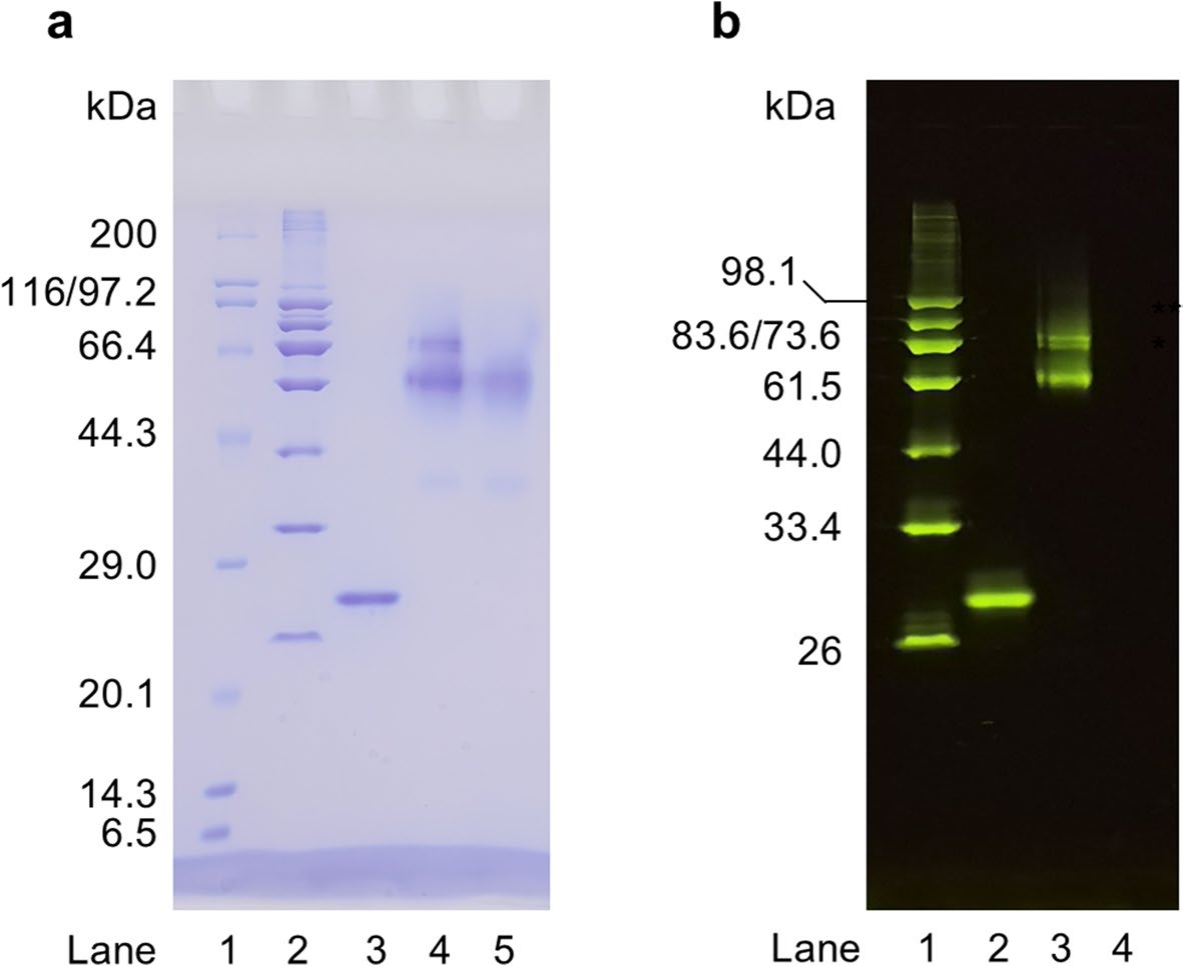
Biotinylation of TGP for sybody selection.** a** SDS-PAGE analysis of TGP biotinylation. The gel was stained using Coomassie staining. Lane 1, broad-range marker with their molecular weight labeled on the left; Lane 2, home-made marker; Lane 3, biotinylated TGP; Lane 4, biotinylated TGP incubated with streptavidin; Lane 5, streptavidin. **b** In-gel fluorescence analysis of TGP biotinylation. Lane 1, home-made fluorescence marker with their theoretical molecular weight labeled on the left; Lane 2, biotinylated TGP; Lane 3, biotinylated TGP incubated with streptavidin; Lane 4, streptavidin

TGP was enzymatically biotinylated at an engineered Avi-tag using BirA enzyme. Labeling efficiency was assessed by a gel mobility shift assay. Upon incubation with streptavidin, both the Coomassie-stained band (Lane 3, Fig. 2a) and the fluorescent band (Lane 2, Fig. 2b) of the BirA-treated TGP completely shifted, with the original bands disappearing and two new bands appearing (Lane 4, Fig. 2a; Lane 3, Fig. 2b). The upper band migrated at approximately 78 kDa, likely corresponding to one TGP molecule (~ 25 kDa) bound to a streptavidin tetramer which runs as a smear band between 52—60 kDa (Lane 5, Fig. 2a). The lower band migrated at approximately 58 kDa, likely representing one TGP bound to a SDS-induced streptavidin dimer.

Following in vitro translation using purified ribosomes, biotinylated TGP was added to the mixture to allow TGP to bind with nascent nanobodies that were still part of a complex with the encoding mRNA and the ribosome. Magnetic streptavidin beads were then added to pull-down the complex. mRNA recovered from the pellet was reverse-transcribed into cDNA and used to construct a phage display library for subsequent selection rounds.

**Fig. 3:**
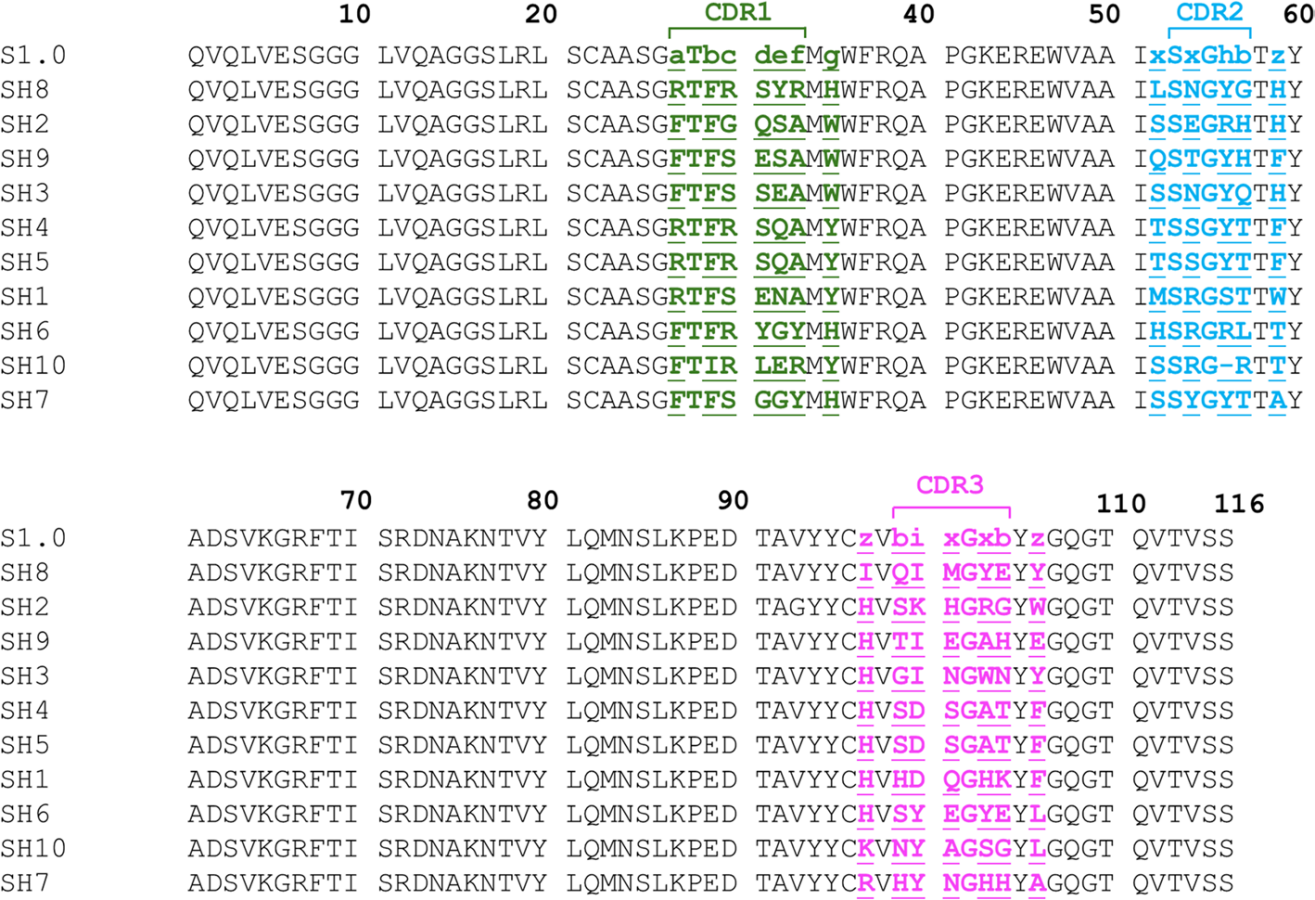
Sequence alignment of the TGP-targeting sybodies from the S1.0 library. CDR and the flanking site with randomization are colored green (CDR1), cyan (CDR2), and magenta (CDR3). Non-capital letters indicate randomized composition as explained in (Fig. 1f). Residues numbers are indicated on the top. Sybodies are arranged by similarity from high (top) to low (bottom) comparing with the S1.0 consensus sequence

The phage library was panned against TGP for two rounds under increasingly stringent conditions, with high concentrations of non-biotinylated TGP in the panning solution to exclude high off-rate binders. Enrichment was monitored by comparing phage binding to TGP-coated beads versus beads coated with a negative control protein (MBP). A semi-quantitative analysis of phage particles showed increasing enrichment (calculated as the ratio of experimental to control group) with an enrichment factor of 427 after the second round of phage display, indicating successful selection of specific binders. Sanger sequencing of 12 colonies revealed 10 unique sequences (Fig. 3).

### Characterization of TGP sybodies

**Fig. 4:**
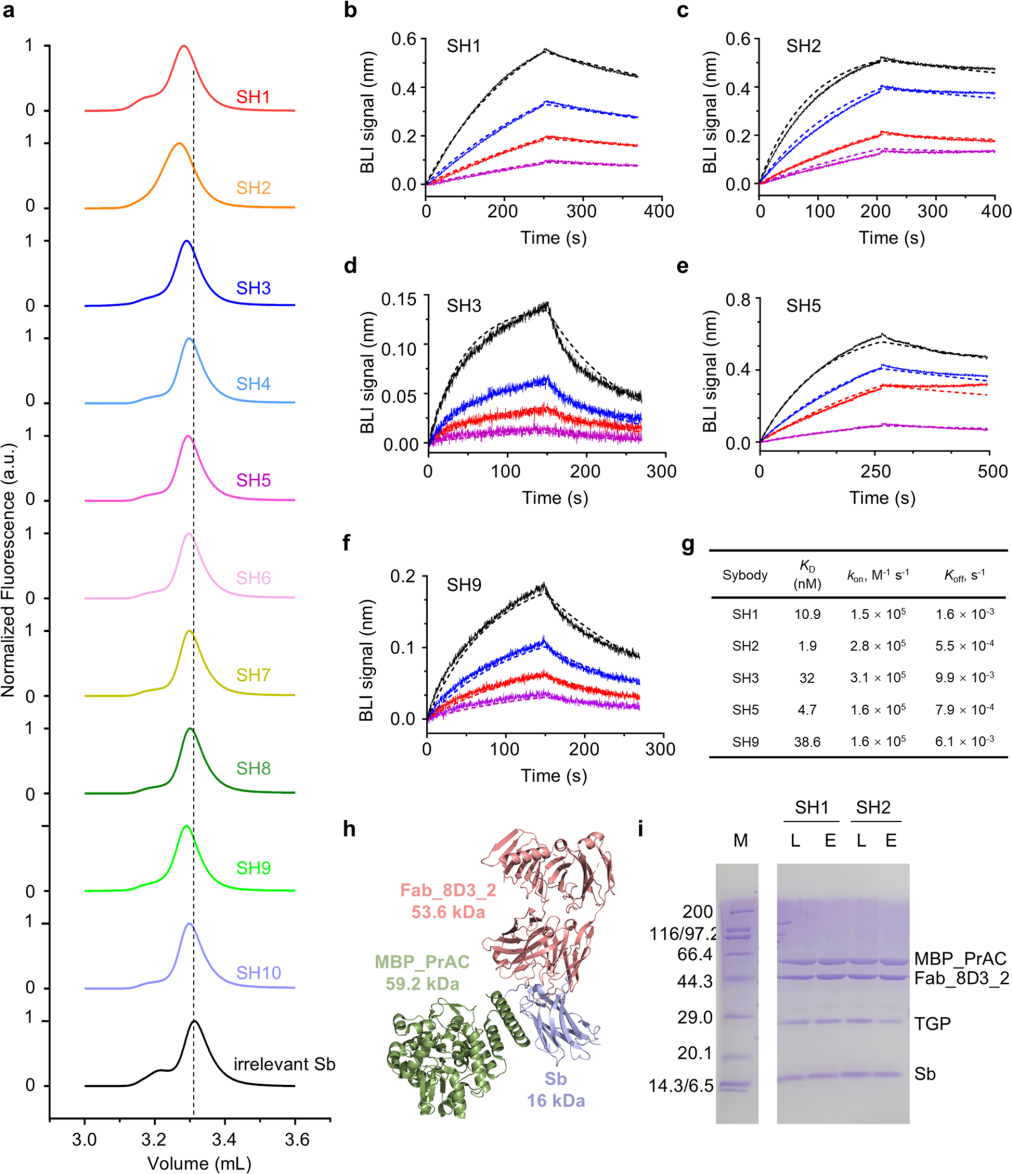
Characterization of the screened sybodies. **a** Identification of TGP binders using FSEC. TGP mixed with sybodies from this study (color) or an irrelevant nanobody (black) were analyzed using FSEC. **b**-**f** Binding kinetics of sybodies with TGP. Biolayer interferometry (BLI) assay was performed with TGP immobilized and sybody as analyte at various concentrations (black, 40 nM; blue, 20 nM; red, 10 nM; purple, 5 nM). Data (solid line) were fitted with built-in software (dotted line) using a 1:1 stoichiometry. **g** Summary of sybody binding kinetics. **h** Structural illustration of the components in the sybody-Legobody assembly (PDB: 7RXC) (Wu and Rapoport 2021). The sybody-recognizing Fab (8D3—2), the engineered maltose binding protein (MBP-PrAC), and the sybody (Sb) are colored red, green, and light blue, respectively. **i** SDS-PAGE analysis of Legobody assembly with S1.0 sybodies. Sybodies were incubated with the Legobody components which contains a MBP protein for binding with amylose resin. The pull-down fraction (E, elution) was loaded alongside of the loading control (L) to compare the band intensities

We expressed the 10 clones and assessed their ability to form complexes with TGP using fluorescence-detection size-exclusion chromatography (FSEC). Leveraging TGP’s intrinsic fluorescence, we screened crude periplasmic extracts without purification by mixing them with TGP before injection onto an analytical HPLC column equipped with a fluorescence detector. All of the ten sybodies exhibited peak shifts compared with TGP incubated with an irrelevant nanobody (Fig. 4a).

We further quantified binding kinetics of a subset of the binders using bio-layer interferometry (BLI) (Fig. 4b-f). Consistent with the observed peak shifts, all characterized sybodies bound TGP with affinities in the nanomolar range. The best binder, SH2, showed a dissociation constant (*K*_D_) of 1.9 nM (Fig. 4g).

### Binding of S1.0 sybodies to Legobody

To investigate whether sybodies selected from the S1.0 library could assemble with Legobody components, pull-down experiments were performed using two representative sybodies, SH1 and SH2. The TGP-bound sybodies were mixed with Legobody components which contains a maltose-binding protein (MBP) (Fig. 4h). After incubation with amylose resin and extensive washing, bound proteins were eluted with maltose. The eluted fractions, along with a loading control (pre-mixed at equimolar ratio for all components), were analyzed by SDS-PAGE. Both SH1 and SH2 were readily detected in the elution fractions (Fig. 4i), demonstrating successful formation of stable sybody–Legobody assemblies in the presence of the antigen TGP.

## Discussion

Nanobodies are increasingly recognized as powerful tools in both basic research and biomedical applications. Synthetic libraries offer several key advantages over animal immunization, including the ability to perform selection under non-physiological conditions, reduced antigen consumption, shorter timeline, and independence from animal facilities. The overall quality of these libraries is critical for successful nanobody discovery. With growing structural data on nanobody-antigen complexes, library design continues to improve. Here, we designed a new library (S1.0) based on structural observations from existing concave sybody complexes.

Validation against two soluble proteins, calmodulin and thermostable green fluorescent protein (TGP), demonstrated that the S1.0 library performs with comparable efficiency and binder quality to the benchmark Seeger Concave library (Zimmermann et al. 2018). Notably, sybodies selected from S1.0 exhibited nanomolar affinities and were directly compatible with the Legobody toolkit without additional engineering. This intrinsic compatibility represents a major practical advantage, as it eliminates subcloning steps and significantly increases throughput for generating binders suitable for cryo-EM studies of small membrane proteins.

Nevertheless, several limitations remain. Our evaluation was performed on only two soluble targets, and the robustness of the S1.0 library against more challenging membrane protein targets has yet to be demonstrated. In addition, while NGS analysis confirmed good overall fidelity to the designed compositions, some positions showed larger deviations than anticipated, which may subtly affect effective library diversity.

For synthetic nanobody libraries, effective functional diversity is more important than theoretical sequence diversity, as the latter is ultimately constrained by the physical limits of the display system (e.g., ribosome or phage copy number). Future library optimization should continue to balance the number of randomized positions with the degree of randomization at each site, guided by both natural amino acid usage in antigen-binding interfaces and empirical selection outcomes. For example, sequencing data from this study revealed that isoleucine at position 29 was strongly underrepresented among functional sybodies, despite being encoded at 20% frequency in the design. Such observations can inform targeted refinements in subsequent library versions.

We anticipate that the increased randomization in CDR1 will shift the paratope distribution compared with the original concave library. This hypothesis can be tested through structural characterization of a larger set of S1.0-derived sybodies. Finally, as large-scale NGS data from naïve and enriched libraries continue to accumulate, data mining and machine learning approaches hold considerable promise for guiding next-generation library design. The establishment of public repositories for raw sequencing data from synthetic nanobody libraries would greatly accelerate progress in this direction.

## Supplementary Information

Supplementary Material 1.

## Data Availability

All data generated or analyzed during this study are included in this published article.
