# Supplementary material for: Design and validation of a ribosome display library for synthetic nanobody selection
Source: Adv Biotechnol (Singap). 2026 May 14;4(2):19. doi: 10.1007/s44307-026-00112-z (PMC13176400; doi:10.1007/s44307-026-00112-z)
Supplement: Supplementary file 1 — Supplementary Material 1. [file 44307_2026_112_MOESM1_ESM.docx]

**Design and Validation of A Ribosome Display Library for Synthetic Nanobody Selection**

Weijie Gu^1,#^, Yaning Li^1,#^, Jingjing Hong^1,#^, Zhihao Yue^2^, Yudi Zhang^1^, Shuting Fan^2^, Zhaowen Shen^2^, Tingting Li^2,*^, Dianfan Li^2,*^

^1^Center for Excellence in Molecular Cell Science, Shanghai Institute of Biochemistry and Cell Biology, University of CAS, Chinese Academy of Sciences, 320 Yueyang Road, Shanghai 200031, China.

^2^School of Agriculture and Biotechnology, Sun Yat-sen University, Shenzhen, China.

^#^Equal contribution

*Correspondence: [litt226@mail.sysu.edu.cn](mailto:litt226@mail.sysu.edu.cn); [lidf9@mail.sysu.edu.cn](mailto:lidf9@mail.sysu.edu.cn)

**Supplementary Information**

Fig. S1

Table S1

**
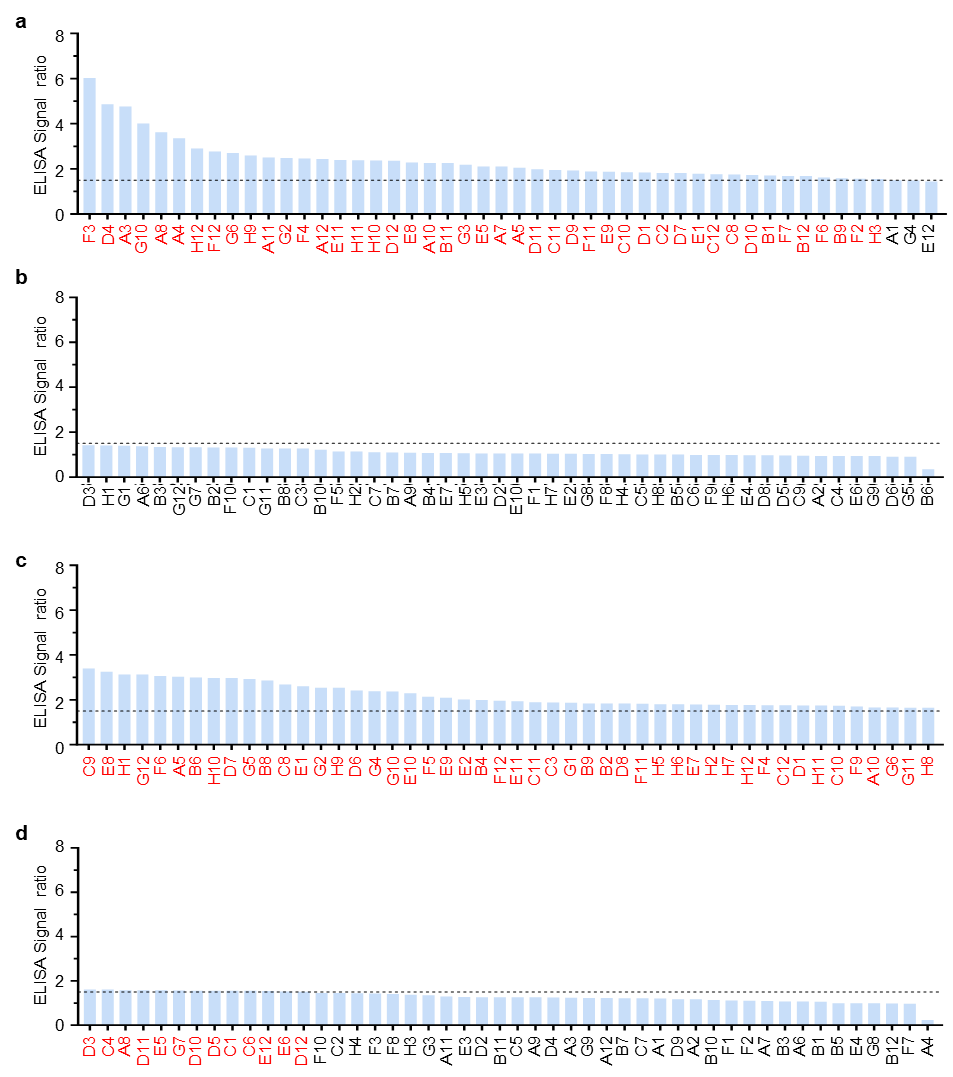
Fig. S1. ELISA identification of potential calmodulin binders. a, b** Results for the S1.0 library. **c, d** Results for the Seeger Concave library. ELISA signals were developed in the presence or absence of Ca^2+^ and the ratio of Ca^2+^-containing condition over the EGTA-containing condition was plotted for each colony. Colonies with ratio over 1.5 (dashed line) are labeled with red texts.

**Table S1. NGS results of the naïve library.**

**
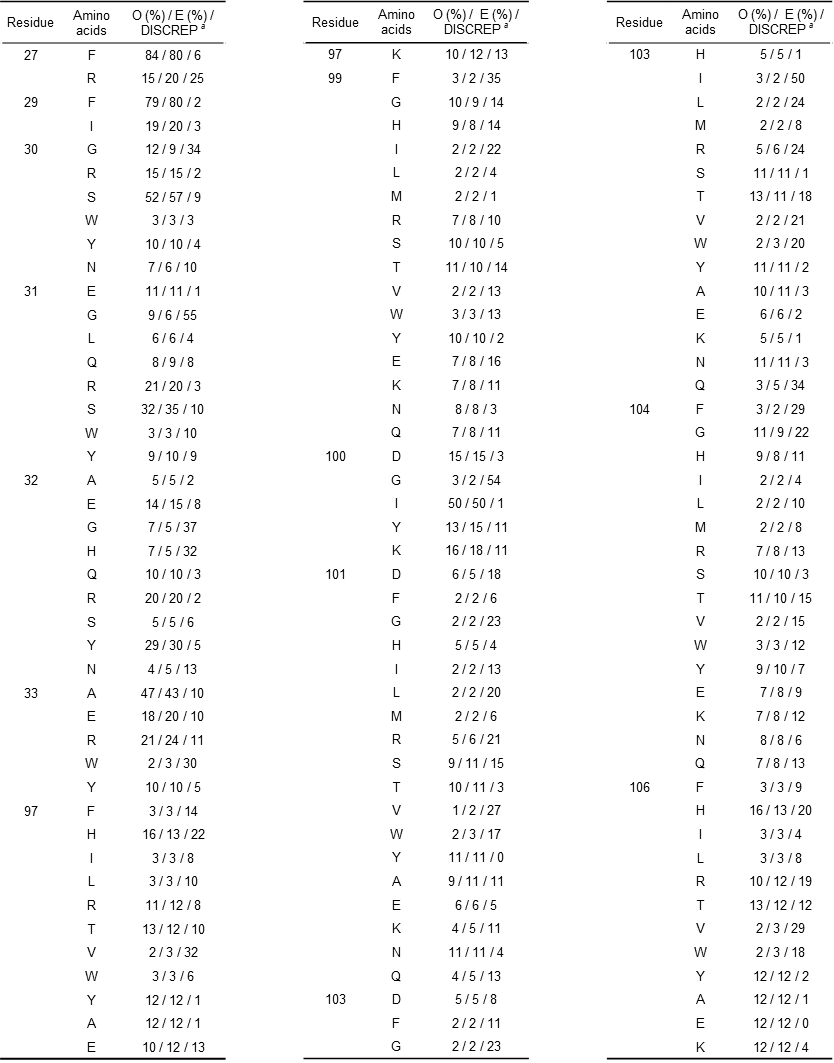
**

*^a^*Abbreviations: O, observed; E, excepted; DISCREP, discrepancy between the observed and the expected value.
